# Enhancing the Solubility and Antibacterial Efficacy of Sulfamethoxazole by Incorporating Functionalized PLGA and Graphene Oxide Nanoparticles into the Crystal Structure

**DOI:** 10.3390/pharmaceutics17111460

**Published:** 2025-11-12

**Authors:** Mohammad Saiful Islam, Indrani Gupta, Edgardo T. Farinas, Somenath Mitra

**Affiliations:** Department of Chemistry and Environmental Science, New Jersey Institute of Technology, Newark, NJ 07102, USA; mi238@njit.edu (M.S.I.); ig72@njit.edu (I.G.); edgardo@njit.edu (E.T.F.)

**Keywords:** antibacterial agent, drug conjugate, enhanced efficacy, poly (lactic-co-glycolic acid), graphene oxide

## Abstract

**Background/Objectives**: The widespread use of sulfamethoxazole (SMX) has led to increasing antibiotic resistance, and there is a need for improved formulations to enhance its therapeutic effectiveness. In this study, we investigated the biocidal potential of SMX composite crystals incorporated with functionalized poly(lactic-co-glycolic acid) (*nf*PLGA) and nano-graphene oxide (nGO). **Methods**: The composites, namely SMX-*nf*PLGA and SMX-nGO, were synthesized via antisolvent precipitation and evaluated using Kirby–Bauer disk diffusion assays. **Results**: Incorporation of *nf*PLGA and nGO significantly improved SMX solubility, increasing it from 0.029 mg/mL to 0.058 mg/mL and 0.063 mg/mL, respectively. Additionally, the log partition coefficient (log P or K_w_) also improved from 1.4 to 0.86 for nGO and 0.92 for *nf*PLGA composites. Both formulations exhibited improved antibacterial activity with distinct time-dependent bactericidal effects. Compared to pure SMX, the SMX-*nf*PLGA showed 60% and 53% greater bacterial inhibition at concentrations of 50 mg/mL and 100 mg/mL, respectively. Although SMX-nGO was slightly less potent, it still surpassed pure SMX, with 50% and 33% higher inhibition at the same concentrations. **Conclusions**: Importantly, neither *nf*PLGA nor nGO showed any biocidal effects, confirming that the observed enhancement was due to improved SMX solubility caused by their incorporation. These findings suggest that embedding solubility-enhancing nanoparticles into the existing crystal structure of the antibiotic is a promising strategy for enhancing the effectiveness.

## 1. Introduction

The discovery of antibiotics in the early 20th century was a major milestone, leading to a significant reduction in illness and death caused by bacterial infections [1,2,3]. However, in recent days, the rise of antibiotic resistance has made it harder to treat infections effectively [4,5,6]. Since developing new antibiotics is a slow, expensive, and difficult process [7,8], there is a need to explore other approaches to improve treatment. These include using drug combinations to increase the effectiveness of existing antibiotics [9,10], and designing better drug delivery systems that can improve the efficacy of antibiotics [11]. Nanomaterials have shown great promise in this area because of their special physical and chemical properties. They can control solubility and bioavailability and help deliver drugs more directly to the target, or improve how cells interact with the drug [12,13].

Various nanocarrier systems, including liposomes, polymeric nanoparticles, and dendrimers, have been studied for antibiotic delivery [13,14,15,16]. For instance, polymeric nanoparticles loaded with ciprofloxacin have shown enhanced antibacterial activity against *Escherichia coli* compared to the pure drug [17]. Nanotechnology-enhanced antibiotics offer several advantages, such as control release for water-soluble drugs, enhanced release for hydrophobic drugs, improved pharmacokinetics, and targeted delivery [18]. Some nanomaterials can even be specifically engineered for easier entry into bacterial cells [19,20]. These innovations can reduce required dosages and lower toxicity [21], making them valuable to antibacterial therapies.

Graphene oxide (GO) has gained significant attention in drug delivery applications owing to its unique physicochemical properties, including a large specific surface area, tunable surface chemistry, electrical conductivity, and inherent biocompatibility. These features enable GO to effectively adsorb and carry therapeutics. The abundant oxygen-containing functional groups on GO surfaces also facilitate further chemical modification, allowing targeted delivery and controlled release profiles, while simultaneously improving drug stability and minimizing premature degradation in physiological environments [22,23]. In parallel, poly(lactic-co-glycolic acid) (PLGA) is an FDA-approved, biodegradable polymer that has been widely employed in pharmaceutical formulations and controlled release systems. By adjusting its lactic-to-glycolic acid ratio and molecular weight, drug release kinetics can be tailored to achieve sustained or delayed therapeutic effects. This controlled release behavior has proven particularly valuable for antibiotics, where maintaining therapeutic concentrations over extended periods can significantly enhance treatment efficacy and suppress the development of antibiotic resistance in pathogenic bacteria [24]. Moreover, the presence of nanocarriers such as these can facilitate targeted delivery to infection sites, reducing off-target toxicity and required dosage. Together, these combined effects may improve drug solubility, stability, and bioavailability, representing a promising strategy for developing next-generation antibiotic formulations that can better overcome microbial resistance mechanisms and improve therapeutic outcomes [25,26].

Sulfamethoxazole (SMZ) is a sulfonamide antibiotic widely prescribed to treat infections caused by both Gram-positive and Gram-negative bacteria [22,23]. Despite its broad-spectrum activity, the extensive use of SMZ over the years has led to the emergence of antibiotic-resistant strains, particularly *Escherichia coli* [24,25,26,27]. *E. coli* is a leading cause of many diseases such as urinary tract infections, and the growing resistance to SMZ is problematic [25,26,27]. This growing threat highlights the urgent need to either develop new antibiotics or reformulate current drugs to improve their therapeutic outcomes.

Recent studies have highlighted the promising role of nanoparticles in enhancing the effectiveness of sulfamethoxazole (SMX) against drug-resistant bacteria [28,29]. The use of metal nanoparticles and metal oxides to enhance the efficacy of sulfamethoxazole involves several mechanisms that significantly improve its antimicrobial activity [30,31]. Metal nanoparticles, such as silver, gold, or copper, exhibit intrinsic antimicrobial properties that can disrupt bacterial cell membranes, leading to cell lysis and death [32,33,34,35]. When combined with sulfamethoxazole, these nanoparticles can create a synergistic effect, enhancing the overall antimicrobial efficacy against resistant strains of bacteria. Moreover, metal nanoparticles can penetrate and disrupt biofilms, protective environments that bacteria form, thereby increasing the effectiveness of sulfamethoxazole against chronic infections [36]. Finally, the presence of metal nanoparticles may help mitigate bacterial resistance mechanisms, further boosting the efficacy of the antibiotic [37].

Improving the solubility of hydrophobic antibiotics such as SMX, which in turn enhances drugs’ bioavailability and absorption in target tissues, can also be an effective approach to enhancing their efficacy. Recently, we have reported the incorporation of nano-functionalized PLGA (*nf*PLGA) and nano-graphene oxide (nGO) into drug crystals to improve the solubility of active pharmaceutical ingredients (APIs) [38,39,40,41,42]. Several APIs, such as griseofulvin, dexamethasone, and apixaban, have demonstrated enhanced dissolution rates and improved initial dissolution rates ranging from 50 to 75 percent. In a previous study, we reported that with incorporated nGO in SMX, the time required for 50% dissolution dropped dramatically from 42 min to 14 min, or a 67% reduction [41]. Importantly, the incorporation of *nf*PLGA and nGO did not alter the crystal structures or physical properties, including the melting point [41].

Sulfamethoxazole is a BCS class IV antibiotic drug with an aqueous solubility of 0.61 mg/mL at 37 °C. It is hypothesized that enhanced dissolution of SMX in the presence of nGO [41] and *nf*PLGA [39] will lead to higher antibacterial activity of SMX. The objective of this study was to test this hypothesis by evaluating whether the incorporation of *nf*PLGA and nGO into sulfamethoxazole (SMX) crystals can enhance its antibacterial efficacy against *E. coli.*

## 2. Experimental Section

### 2.1. Materials, Chemicals, and Methods

*E. coli* C-3000 strain (ATCC-15597) was purchased from American Type Culture Collection (ATCC), VA, USA. Sulfamethoxazole (SMX) was purchased from Sigma Aldrich (St. Louis, MO, USA). PLGA was bought from Poly Sciences Inc. (Warrington, PA, USA). Acetone was bought from Sigma Aldrich. Sulfuric acid, nitric acid, hydrochloric acid, and acetone were purchased from Fisher Scientific (Thermo Fisher Scientific Inc., Waltham, MA, USA). Graphene oxide (GO) dispersion sheets (42–52% carbon) were purchased from Sigma Aldrich. Acetonitrile was purchased from Sigma Aldrich. Disk diffusion kits were obtained from Flinn Scientific (Batavia, IL, USA). The water used in the experiment was purified with the Milli-Q plus system at the York Center facility, NJIT (Newark, NJ, USA).

### 2.2. Bacterial Propagation

A single colony of *E. coli* is obtained by streaking the cells on a Luria broth (LB) agar plate, and the cells are incubated for 24 h at 37 °C. The colony is then inoculated into 15 mL of LB culture and incubated for 18 h at 37 °C. The resulting culture is then inoculated into 100 mL of LB media and incubated at 37 °C and 250 RPM until the optical density (OD600 nm) reaches its initial log phase (OD 0.4–0.6). The culture is then divided into equal amounts for experimental analysis.

### 2.3. Synthesis of SMX-nfPLGA

An antisolvent technique was used to precipitate SMX-*nf*PLGA composites. A predetermined amount of sulfamethoxazole (SMX) was weighed and dissolved in acetone, and a clear solution of *nf*PLGA was also made in acetone in a separate container. This was added to the SMX solution gradually, dropwise, and bath-sonicated for 10 min to ensure complete dissolution and a homogeneous mixture. The concentration of *nf*PLGA was selected based on the desired drug-to-polymer ratio for this composite. Next, the composite solution was placed into a cold ice bath to lower the temperature, and antisolvent milli-Q water was added dropwise, causing the SMX-*nf*PLGA composites to precipitate. The milky suspension of drug–polymer composites was observed during the precipitation process. The solid precipitate was then filtered, transferred, and dried in a vacuum oven at 60 °C to maintain a constant dry weight. Next, the dried SMX-*nf*PLGA composites were stored in a moisture-proof glass container until further use or analysis.

### 2.4. Synthesis of SMZ-nGO

An antisolvent precipitation was also used for nGO incorporation into the drug crystals. First, a colloidal suspension of graphene oxide (nGO) at a concentration of 2% (*w*/*v*) in milli-Q water was prepared by fully dispersing it by probe ultrasonication for 60 min. A solution of sulfamethoxazole (SMX) was prepared in an acetone solvent. Next, a colloidal suspension of nGO was added dropwise to the SMX drug solution over 10 min under bath ultrasonication to facilitate the uniform mixing and enhance the interaction between nGO and SMX. Next, Mill-Q water was added to the SMX-nGO mixture, which slowly generated the cloudy suspensions of crystals. The resulting mixture was kept in a fume hood to reach room temperature. When the precipitation was complete, it was filtered through a membrane filter to separate the solid composite (designated as SMX-nGO) from the liquid phase. The precipitate was washed multiple times to remove residual solvents and impurities, and the pH of the composite was adjusted to 7.0. Finally, the washed SMX-nGO composite was transferred to a vacuum oven and dried under a vacuum pressure of 30.0 mm-Hg for 48 h until a constant weight was achieved, indicating complete removal of moisture.

### 2.5. Antibacterial Studies

Bacterial viability was studied using Kirby–Bauer disk diffusion assays. In the disk diffusion assay, 20 µL of dissolved SMX/SMX conjugates was loaded onto the disk, placed on *E. coli* (OD600 of 0.5) LB agar plates, and incubated overnight at 37 °C. In Table 1, the concentrations of pure SMX/SMX conjugates that were loaded onto the disks are listed. The growth-inhibitory zone around the disks was obvious in the bacterial inhibition zone assays. The diameter of the zone of inhibition (D) was measured from one end to the other end of the clear zones and was subtracted from the diameter of the disks. Finally, the highest amount of antibacterial activity was defined as 100% activity, and other amounts were compared to it.

### 2.6. Drug Formulation Property Analysis

A JEOL JSM-7900F scanning electron microscope (SEM) (JEOL, Tokyo, Japan) was used to capture images of the drug crystals. Powder X-ray diffraction (PXRD) was conducted to verify the crystalline identity of the SMX drug formulations using the PANalytical EMPYREAN XRD (Malvern, UK) instrument with a Cu Kα radiation source. The diffraction intensity was measured over a 2θ range of 5–70°. Raman spectral intensity was measured using a ThermoFisher Scientific DXR2xi Raman imaging microscope instrument (Madison, WI, USA), which employed a 532 nm laser wavelength and a full-frequency acquisition mode spanning the 3800–200 cm^−1^ region. Fourier-transform infrared (FTIR) analysis was performed (using diamond ATR) with an Agilent Cary 670 Benchtop spectrometer (Santa Clara, CA, USA) to assess the functional properties of the drugs. The melting points of the SMX-nGO/nfPLGA drugs were measured using a Differential Scanning Calorimeter (PerkinElmer DSC 6000, Shelton, CT, USA). The DSC analysis operated between 30 and 300 °C at 10 °C/min, with a sample amount of 5–10 mg. The drug aqueous concentration, solubility, and concentration in the octanol–water partitioning phase were determined by using a UV-Vis spectrometer, Thermo Scientific, Waltham, MA, USA.

## 3. Results and Discussion

### 3.1. Material Characterization 

#### 3.1.1. Morphology and Physicochemical Property Analysis

**Figure 1:**
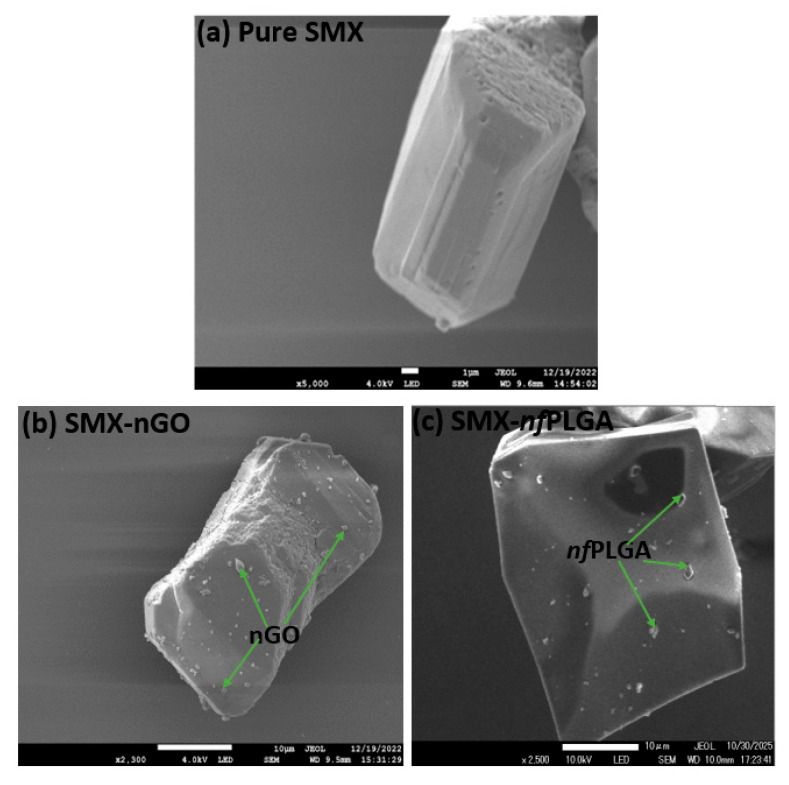
Scanning electron microscopy (SEM) images of (**a**) pure SMX, (**b**) SMX-nGO, and (**c**) SMX-*nf*PLGA.

SMX-*nf*PLGA and SMX-nGO were successfully formulated by using the antisolvent approach. The scanning electron microscopy (SEM) images of pure SMX, SMX-nGO, and SMX-*nf*PLGA composites are presented in Figure 1a–c. The SEM images of the composite crystals (Figure 1b,c) show the presence of nGO in Figure 1b and *nf*PLGA in Figure 1c on the drug crystal surface, and also indicate that the incorporation of *nf*PLGA or nGO did not alter the crystal structure. The sizes of the nGO and *nf*PLGA were analyzed using dynamic light scattering (DLS) and were found to be 150 and 160 nm, respectively [39,43]. The presence of hydrophilic functionalized *nf*PLGA or nGO on the crystal surfaces led to hydrophilic interactions with water molecules, creating hydrogen bonds that generated channels for enhanced aqueous solubility and dissolution performance. Our previous studies with other APIs have demonstrated not only higher dissolution but also faster dissolution [38,39,40,41].

Aqueous solubility at room temperature is an important parameter for the drug formulations. SMZ is hydrophobic (BCS-IV drug) and insoluble in aqueous media. The incorporation of nGO increased the water solubility of SMX-nGO to 0.063 mg/mL and *nf*PLGA increased the water solubility of SMX-*nf*PLGA to 0.058 mg/mL in 24.0 h (Table 2). Pure SMZ did not dissolve or partially disperse, whereas the composites did dissolve to the maximum to reach a saturated concentration. It is clear from the data that as nGO or *nf*PLGA is incorporated, solubility increases. This occurs because the nGO or *nf*PLGA dispersed around the drug crystals helps channel water through hydrogen bonding.

A 1-octanol/water partitioning study was conducted to provide further insight on physicochemical properties of formulated composites which could affect bioactivity such as absorption, distribution, metabolism, and excretion. The logP value obtained from this experiment, also known as the partitioning coefficient, is a measure of hydrophobicity. It correlates with oral drug solubility and interacts with the phospholipid membrane. The calculated logP values for the SMX-nGO and SMX-*nf*PLGA composites were 0.86 and 0.92, respectively, compared to 1.40 for pure SMX (Table 2). This suggested that the SMX composites were less hydrophobic than the pure drug. Hence, their ability to dissolve in the aqueous layer was reduced.

Enhanced solubility can improve absorption and bioavailability, boosting the drug’s effectiveness at the target site. Additionally, incorporating nanomaterials such as graphene oxide and PLGA nanoparticles may further enhance the antibacterial effectiveness of SMX by increasing the effective drug concentrations at infection sites. The octanol–water partitioning values (logP) of the SMX composites are also a reflection of this enhancement, as a lower logP correlates with improved solubility and leads to higher cellular uptake, ensuring that higher concentrations of the drug reach the site of infection. The modifications made with these composites demonstrate potential for advancing drug delivery systems and enhancing therapeutic outcomes against resistant bacterial strains.

**Figure 2:**
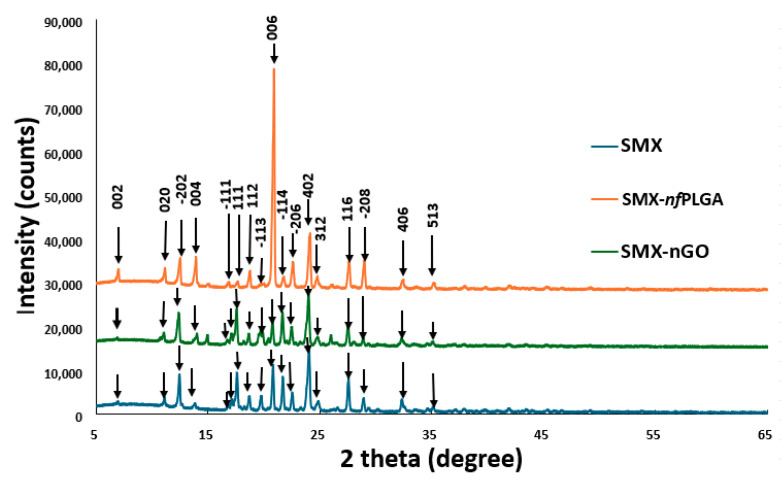
X-ray powder diffraction (XRD) analysis of the SMX composites.

The incorporation of *nf*PLGA or nGO into the SMX drug was carefully evaluated concerning its crystallinity and polymorphism. X-ray powder diffraction analysis, depicted in Figure 2, shows the major 2θ peaks of pure SMX at approximately 12°, 15°, 18°, 21°, 22°, 25°, 27°, and 30°. The XRD patterns indicate that the same Miller indices for triclinic SMX (e.g., 002, 020, 111 at 7–25°) are present in pure SMX, as well as in the SMX-*nf*PLGA and SMX-nGO composites. Notably, FCC indices are negligible, and no shifts in peak positions were detected. This evidence supports the conclusion that the polymorphism of SMX remains unchanged. Additionally, the presence of consistent peaks in both SMX-*nf*PLGA and SMX-nGO nanocomposites demonstrates that the crystalline structure of SMX has been largely preserved, despite the addition of nanoparticles. The retention of peak positions across all samples underscores the successful integration of SMX into the nanocomposites.

**Figure 3:**
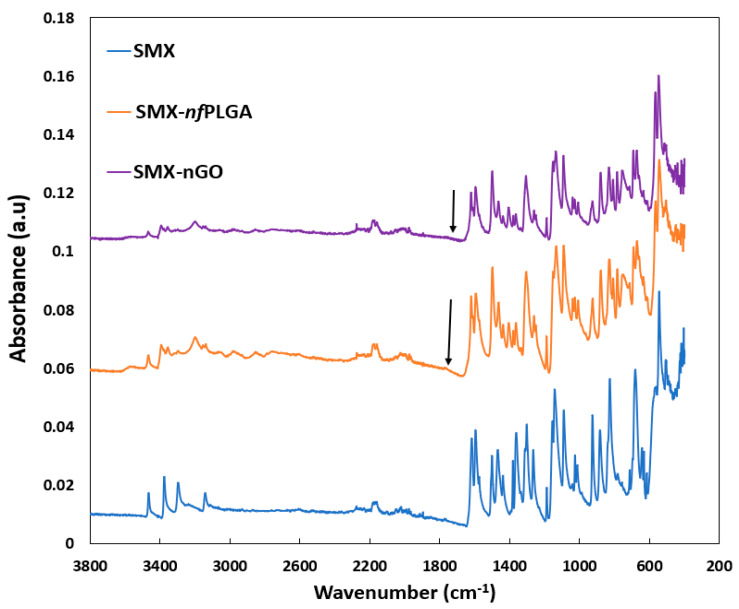
Fourier-transform infrared spectroscopy (FTIR) analysis of the SMX composites.

The FTIR analysis Figure 3 indicates the characteristic absorption bands of pure SMX, including prominent peaks around 3400 cm^−1^ (O-H/N-H stretching), 1650 cm^−1^ (C=O stretching), 1600 cm^−1^ (aromatic C=C), and 1150 cm^−1^ (S=O stretching). These are also present in both SMX-nfPLGA and SMX-nGO nanocomposites, suggesting that the molecular structure of SMX remains largely intact despite incorporation. This consistent retention of key absorption features across all samples underscores the successful integration of SMX into the nanocomposites, potentially improving its solubility. The FTIR spectra show SMX (blue) with dominant peaks at ~3300–3500 cm^−1^ (N–H), ~1600 cm^−1^ (aromatic C=C), and ~1300–1150 cm^−1^ (S=O); SMX-*nf*PLGA (orange) retains SMX peaks with added *nf*PLGA C=O at ~1768 cm^−1^; and SMX-nGO (purple) displays SMX features alongside broad GO O–H (~3400 cm^−1^) and C=O (~1744 cm^−1^), confirming successful incorporation of *nf*PLGA and nGO without altering SMX’s core functional groups.

**Figure 4:**
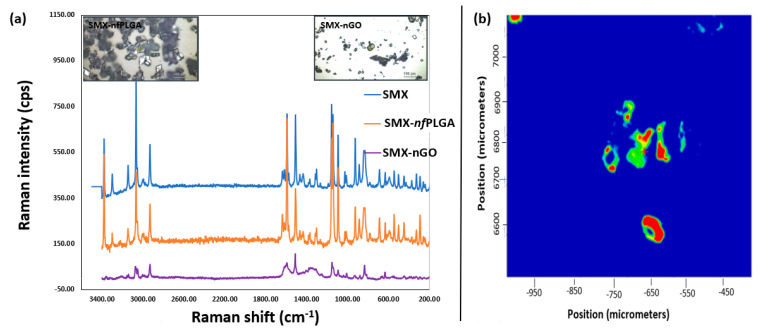
(**a**) Raman spectra and (**b**) mapping and imaging analysis of the SMX drug composite crystals.

Additionally, Figure 4a presents the Raman spectrum of SMX, SMX–*nf*PLGA, and SMX-nGO composites after the nanomaterial incorporation. The spectral intensity for the composites shows no variation in the peaks associated with the different functional groups. Also, as presented in Table 2, the melting point also remained unchanged. Therefore, it is concluded that there was no variation in polymorphism. As a result, SMX in the SMX-*nf*PLGA or SMX-nGO composites is expected to remain biologically similar with increased solubility through the incorporation of inactive *nf*PLGA or nGO particles.

Additionally, the Raman mapping and imaging in Figure 4b was carried out to see the distribution of the nanoparticle (*nf*PLGA/nGO) on the single-drug crystal surface. The distribution map for the SMX-*nf*PLGA composites showed a strong peak at 1660 cm^−1^, corresponding to the carbonyl (C=O) band of the *nf*PLGA-incorporated drug composite [44]. In the Raman image, the bright yellow and red areas represent high SMX concentrations, while the green suggests moderate Raman scattering, implying a transitional zone [45].

#### 3.1.2. Antibacterial Assays

**Figure 5:**
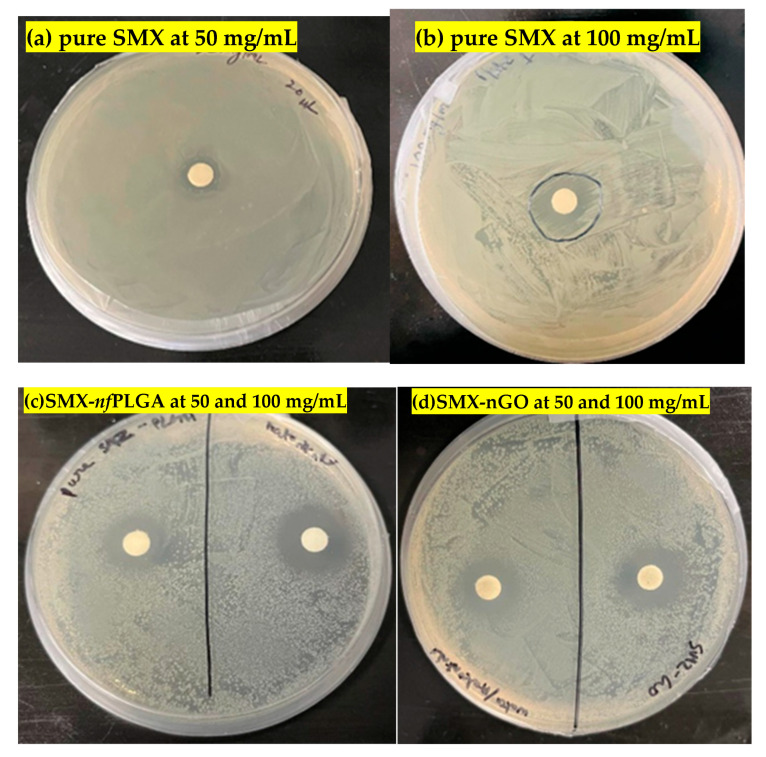
Bacterial inhibition zone assays of (**a**) pure SMX at 50 mg/mL, (**b**) pure SMX at 100 mg/mL, (**c**) SMX-*nf*PLGA at concentrations of 50 and 100 mg/mL, and (**d**) SMX-nGO at concentrations of 50 and 100 mg/mL.

Enhanced bacterial reduction was observed through disk diffusion assays and is presented in Figure 5. Table 3 shows the inhibition zone measurements, and it is evident that upon conjugation with *nf*PLGA, the bactericidal properties of the pure SMX improved by 60% and 53% at 50 mg/mL and 100 mg/mL, respectively. Compared to SMX-*nf*PLGA, SMX-nGO exhibited a slightly lower clear zone (lower by 6.25% and 13.04% at 50 mg/mL and 100 mg/mL, respectively) but delivered a greater efficacy than pure SMX by 50% and 33% at the same concentrations. These values were statistically significant at a confidence level of 98%. Although *nf*PLGA or nGO by itself has no antimicrobial properties, the presence of *nf*PLGA and nGO nanoparticles on the crystal surface improved the solubility and the rate of solubilization and improved the intracellular uptake of antibiotics by target cells of the bacteria. Therefore, the incorporation of *nf*PLGA into the low-solubility antibiotic has the potential to improve the efficacy and can be effective at a lower dose, leading to lower toxicity.

The octanol–water partition coefficient (logP) plays a central role in determining the efficacy of water-insoluble drugs because it governs the balance between solubility and membrane permeability. Drugs with lower logP values may dissolve well in aqueous media, but may have problems crossing the lipid-rich biological membranes, resulting in poor absorption and low efficacy. Even though SMX-nGO had higher solubility, it showed slightly lower efficacy than SMX-*nf*PLGA because of its slightly higher logP, which may have been optimum for biocidal activity against *E. Coli* [41].

Besides logP, PLGA and GO differ significantly in their chemical structures. PLGA is a biodegradable polyester composed of lactic and glycolic acid monomers linked through ester bonds, giving it a largely hydrophobic backbone with moderate lipophilicity. In contrast, graphene oxide contains abundant oxygen-containing groups such as hydroxyl, epoxy, and carboxyl moieties distributed across its surface. These polar functionalities render GO highly hydrophilic and dispersible in water, although it retains some amphiphilic character due to its extended sp^2^-hybridized carbon domains, which enable π–π stacking with aromatic drugs. Overall, PLGA is more lipophilic than graphene oxide, which is more hydrophilic and interacts primarily through surface functional groups rather than through lipid-like partitioning. This fundamental difference also influences biocidal properties and explains the difference between the two composites.

In summary, the incorporation of nGO and *nf*PLGA into SMX crystals offers unique advantages by enhancing the solubility of the BCS class IV, relatively insoluble drug to influence their overall therapeutic potency. The findings of this study highlight the enhanced antibacterial efficacy of sulfamethoxazole (SMX) when incorporated with functionalized, hydrophilic nanoparticles that enhance their solubility. Since the particles themselves did not show any antibacterial activities, it is concluded that the enhancement in antibacterial properties came mainly from their enhanced solubility.

## 4. Conclusions

The antibacterial activity of SMX, SMX-*nf*PLGA, and SMX-nGO was assessed using the Kirby–Bauer disk diffusion method. Clear zones of inhibition were observed around disks loaded with SMX and its nanoparticle conjugates, indicating effective antibacterial properties. This study demonstrates that incorporating functionalized poly(lactic-co-glycolic acid) (*nf*PLGA) and nano-graphene oxide (nGO) into sulfamethoxazole (SMX) significantly enhances its antibacterial efficacy. The synthesized composites, SMX-*nf*PLGA and SMX-nGO, showed improved solubility, with SMX-*nf*PLGA exhibiting notably greater bacterial inhibition compared to pure SMX. These findings suggest that incorporating hydrophilic nanoparticles into the BCS class II crystal can effectively improve existing antibiotic formulations. Future research should focus on in vivo experiments to validate clinical relevance and potential variability in composite performance across biological environments, which would translate these promising results into effective treatments for antibiotic-resistant infections.

## Figures and Tables

**Table 1:** Concentration of materials used in antibacterial studies.

Sl No.	Materials	Material Concentration	
		Disk Diffusion Assays (mg/mL)	O.D. Measurements (µg/mL)
1	Pure SMX	50, 100	100
2	Pure PLGA	-	100
3	Pure GO	-	100
4	SMX-*nf*PLGA	50, 100	100
5	SMX-nGO	50, 100	100

**Table 2:** Physicochemical properties of sulfamethoxazole formulations.

Sulfamethoxazole (SMX) Formulations	Aqueous Solubility (25 °C) (mg/mL)	Partitioning LogP	Melting Temp (°C)
Pure SMX	0.029	1.40	172.51
SMX-nGO	0.063	0.86	170.11
SMX-*nf*PLGA	0.058	0.92	171.14

**Table 3:** Results of the disk diffusion assay.

Serial No.	Concentration (mg/mL)	Inhibition Zone (mm)
**Pure SMX**		
1	50	10
2	100	15
**SMX-*nf*PLGA**		
3	50	16
4	100	23
**SMX-nGO**		
5	50	15
6	100	20

## Data Availability

The data presented in this study are available on request from the corresponding author.
